# Comparison of Clinical Outcomes Between Primary and Salvage Reverse Shoulder Arthroplasty for Proximal Humeral Fractures: A Retrospective Study

**DOI:** 10.1111/os.70018

**Published:** 2025-04-13

**Authors:** Qing Zhang, Sujan Shakya, Yi Cao, Ming Xiang, Zhou Xiang, Xin Duan

**Affiliations:** ^1^ Department of Orthopedic Surgery School of Medicine, West China Hospital, Sichuan University Chengdu China; ^2^ Department of Upper Limb Sichuan Provincial Orthpaedics Hospital Chengdu China; ^3^ Affiliated Hospital of Chengdu University of Traditional Chinese Medicine Chengdu China; ^4^ Department of Orthopedics The Fifth People's Hospital of Sichuan Province Chengdu Sichuan China; ^5^ Department of Orthopedics Ganzi Tibetan Autonomous Prefecture People's Hospital Ganzi Sichuan China

**Keywords:** clinical outcomes, failed osteosynthesis, proximal humeral fracture, reverse shoulder arthroplasty

## Abstract

**Objective:**

This study provides a comparative analysis of clinical outcomes between primary and salvage reverse shoulder arthroplasty (RSA), offering valuable insights into the management of proximal humerus fracture (PHF). To evaluate the outcomes of patients treated with RSA as a primary procedure for acute PHF and to compare these with patients undergoing salvage RSA as a revision procedure for fracture sequelae of PHF.

**Methods:**

A retrospective cohort study was conducted on 42 patients undergoing RSA for PHF between December 2014 and April 2022. The primary RSA group (*n* = 28, mean age 73.8 ± 4.5 years, 66–81 years) included patients with acute fractures, while the salvage RSA group (*n* = 14, mean age 62.1 ± 12.3 years, 47–83 years) comprised revision cases for fracture sequelae. Active range of motion (ROM), Visual Analog Scale (VAS), Constant score, and American Shoulder and Elbow Surgeons (ASES) scores were assessed for all patients. Outcomes between the two groups were compared, along with radiographic outcomes and complications recorded at each follow‐up. Categorical variables were analyzed using chi‐square or Fisher's exact tests, while continuous variables were compared using independent *t*‐tests or Mann–Whitney U tests based on data distribution.

**Results:**

At a mean follow‐up of 56 months (24–106 months), no significant differences in gender (*p* = 0.469) or follow‐up duration (*p* = 0.087) were observed. The salvage group exhibited comparable postoperative ROM (anterior flexion (AF): 101.4° ± 52.3° vs. 115.9° ± 29.1°; external rotation (ER): 26.4° ± 16.4° vs. 28.8° ± 14.1°; internal rotation (IR): 7 ± 2 vs. 7 ± 2; all *p* > 0.05) and clinical scores (VAS: 1.6 ± 1.9 vs. 1.2 ± 1.5; Constant: 74.1 ± 23.3 vs. 79.4 ± 15.9; ASES: 81.9 ± 15.4 vs. 84.0 ± 13.8; all *p* > 0.05) to the primary group. However, the salvage group demonstrated significant preoperative‐to‐postoperative improvements in AF (50.9°, *p* < 0.001), ER (5.4, *p* = 0.017), and functional scores (VAS: −4.6; Constant: + 36.9; ASES: + 45.8; all *p* < 0.05). Complications occurred in 14.3% of salvage cases (2 revisions for periprosthetic fracture and aseptic loosening) versus 3.6% in the primary group. No other major complications such as deep infection, instability, acromial stress fracture, or dislocation were recorded.

**Conclusion:**

RSA achieves comparable functional and radiographic outcomes for both acute PHF and fracture sequelae over 4 years of follow‐up. Salvage RSA provides substantial clinical improvement but carries a higher complication risk, emphasizing the need for meticulous surgical technique and patient selection.

## Graphical abstract

Compare outcomes of primary RSA (acute PHF) versus salvage RSA (fracture sequelae). Retrospective analysis of 42 patients (28 primary, 14 salvage) with > 2‐year follow‐up.

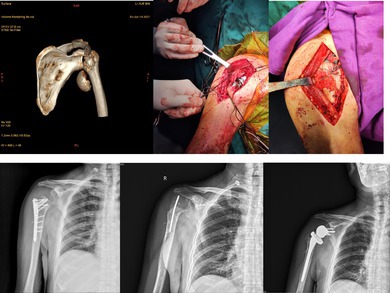

## 1 Introduction

Proximal humeral fractures (PHF) are prevalent among elderly individuals, accounting for approximately 5% of all fractures [1]. While surgical intervention is recommended for only about 20% of cases [2], the surgical management of displaced osteoporotic PHF poses a significant challenge for surgeons in selecting the appropriate treatment method.

When surgical treatment is warranted, options include open reduction and internal fixation (ORIF), hemiarthroplasty, total shoulder arthroplasty, and reverse shoulder arthroplasty (RSA). ORIF is often the first‐line treatment for displaced fractures; however, complications such as nonunion, malunion, intraarticular screw penetration, and osteonecrosis are frequently reported, particularly in elderly patients with severe osteoporosis [3, 4, 5, 6].

Complications arising from failed internal fixation can lead to severe dysfunction and pain due to fracture sequelae [3, 4, 5, 7]. Additionally, shoulder misalignment following malunion can result in the loss of rotator cuff centering, significantly impacting shoulder function. Furthermore, deltoid contraction can cause displacement of the proximal humerus. These issues present numerous challenges for surgeons performing salvage surgery. Salvage procedures for these complications, such as anatomic hemiarthroplasty or total shoulder arthroplasty, are technically demanding and are associated with higher complication rates and inferior outcomes compared to primary arthroplasty [8, 9]. A previous study has shown that 28%–85% of patients undergoing salvage hemiarthroplasty or total shoulder replacement have a poor prognosis [10].

Historically, hemiarthroplasty was the sole option for PHF when arthroplasty was considered. However, its outcomes remain inconsistent and unpredictable [11]. Currently, as alternatives to ORIF and hemiarthroplasty, some surgeons are turning to RSA for complicated PHF cases. RSA has emerged as a promising alternative for complex PHF, particularly in elderly patients [12]. Recent comparative studies have suggested that RSA outcomes are even superior to those of ORIF and hemiarthroplasty for the elderly [13].

RSA has demonstrated efficacy in treating acute PHF [14], symptomatic nonunion, and malunion in elderly patients [3, 7, 15], particularly in cases with rotator cuff deficiency. Given the promising results, indications for RSA have been extended to similar non‐functional rotator cuff conditions, such as sequelae of fractures [16, 17]. RSA has emerged as an effective option to revise failed hemiarthroplasty [5, 6, 18, 19]. Despite these advancements, comparative studies on primary versus salvage RSA outcomes are sparse. Existing literature suggests comparable functional results but higher complication rates in salvage RSA [1, 15, 20], while other reports favor primary RSA [21]. Very few studies have reported RSA for posttraumatic sequelae of PHF with small case series [15, 20]. Whether previous surgery will compromise RSA in the future still needs to be clarified. The lack of sufficient evidence precludes drawing useful clinical conclusions and definitive guidelines for the treatment of fracture sequelae.

The objectives of this study are to: (i) report the efficacy of RSA for acute PHF; (ii) present clinical and radiographic outcomes following salvage RSA for fracture sequelae; (iii) compare clinical and radiographic outcomes between primary and salvage RSA cohorts; and (iv) analyze complications and strategies for avoidance.

## 2 Methods

### 2.1 Patients and Materials

We conducted a retrospective review of patients undergoing primary RSA for acute PHF or salvage RSA for fracture sequelae at our institution between December 2014 and April 2022.

The primary RSA is indicated for patients aged 65 and above with four‐part PHFs with dislocation, or patients aged 70 and above with three‐part or four‐part proximal humerus fractures with severe osteoporosis (BMD < −2.5). The primary RSA is also considered for patients with low functional demand or when there is a significant loss of rotator cuff function, which precludes the success of conventional shoulder replacement or repair techniques.

The salvage RSA is indicated for patients with complications following initial surgical interventions for PHF, including nonunion, malunion, aseptic loosening, infection, or failure of internal fixation leading to significant dysfunction and pain.

#### 2.1.1 Inclusion Criteria

Patients were included if they: (i) sustained PHF and were treated with primary RSA for acute fractures or salvage RSA for fracture sequelae; (ii) had complete clinical data with a minimum follow‐up period of 2 years.

#### 2.1.2 Exclusion Criteria

Patients were excluded if they had: (i) pathological fractures; (ii) prior ipsilateral shoulder surgery; (iii) dementia; (iv) preexisting axillary nerve or deltoid muscle injury.

A total of 42 patients (28 primary RSA, 14 salvage RSA) met the inclusion criteria. All surgeries were performed by a single senior surgeon to minimize technical variability. Each patient had a follow‐up period of over 2 years.

This study was conducted in accordance with the Declaration of Helsinki and was approved by the Ethics Committee of Sichuan Provincial Orthopedic Hospital (approval number: KY2023‐005‐01). Written informed consent was obtained from all participants involved in the study.

#### 2.1.3 Radiographic Evaluation

**FIGURE 1:**
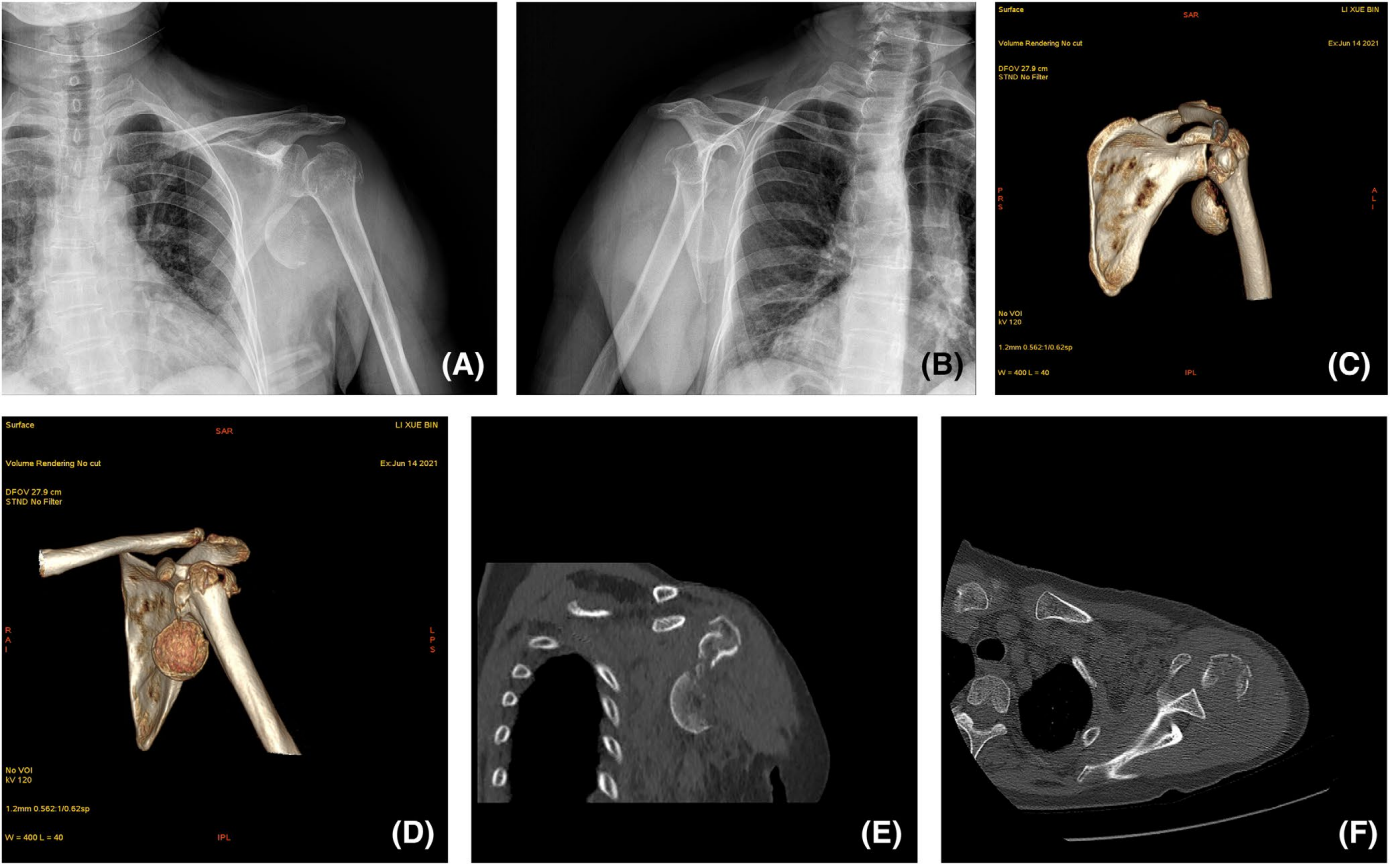
A 72‐year‐old female with left PHF (Neer classification: Four‐part fracture associated with shoulder dislocation) as a result of a fall from stairs. (A, B) Anteroposterior and lateral radiographs demonstrating involvement of the greater and lesser tuberosities with glenohumeral dislocation. (C–F) 3D‐CT reconstruction illustrating fracture displacement and configuration.

Radiographic assessment included standard anterior–posterior and lateral views (Figure 1A,B). Additionally, all patients underwent 3D computed tomography (CT) scans to facilitate a more comprehensive evaluation of the fracture morphology and alignment (Figure 1C–F).

### 2.2 Surgical Methods

All operations were conducted under general anesthesia, supplemented with brachial plexus anesthesia. Patients were positioned in the beach‐chair position, with the surgical extremity appropriately draped and free.

The delto‐pectoral interval approach was utilized for all patients in both groups. The cephalic vein was typically exposed and retracted medially. The deltoid and pectoral muscles were exposed and protected. The interval between the deltoid and pectoralis muscles was developed to expose the greater and lesser tuberosities. Following the exposure of the intertubercular sulcus and dissection of the rotator cuff interval between the supraspinatus and subscapular tendons, the long head of the biceps tendon was identified and detached from its glenoid insertion. The tendinous portion of the rotator cuff was tagged with No. 5 Ethibond nonabsorbable sutures (Ethicon, USA) to facilitate manipulation of the greater and lesser tubercles, thereby exposing the humeral head. An osteotomy was performed, and the humeral head was subsequently excised. Retractors were employed to fully expose the glenoid. The supraspinatus tendon was routinely excised.

In the salvage group, prior implants were removed during one‐stage RSA if no signs of infection were detected. For patients with infection, a two‐stage revision procedure was implemented. The initial step involved the removal of the existing implant, thorough debridement, and insertion of a temporary antibiotic‐impregnated cement prosthesis. The patient was administered a standardized antibiotic regimen for 6 weeks, followed by RSA 3–6 months later. Intraoperative cultures were obtained in all cases, and the final prosthesis was inserted only after confirmation of negative cultures. Both groups received second‐generation Grammont‐style prostheses (Comprehensive Reverse Shoulder System, Zimmer Biomet, USA; Equinoxe Reverse System, Exactech, USA).

After dislocation of the shoulder joint and removal of the humeral head, the glenoid was fully exposed using Hohmann shoulder retractors. The labrum was subsequently resected. A glenoid guide handle was firmly attached to the glenoid, followed by the insertion of a guide needle into the glenoid. A cannulated baseplate reamer was positioned atop the guide column, and all cartilage and scar tissue on the glenoid were meticulously debrided. The glenoid was reamed down to the subchondral bone. Then, the baseplate was inserted and secured with a 6.5 mm central screw and four 4.75 mm peripheral screws. A glenosphere trial of the appropriate size was selected and assembled with a trial taper adaptor. The glenosphere, typically 36 mm in diameter, was then engaged with forceps and implanted in the same orientation as the trial.

**FIGURE 2:**
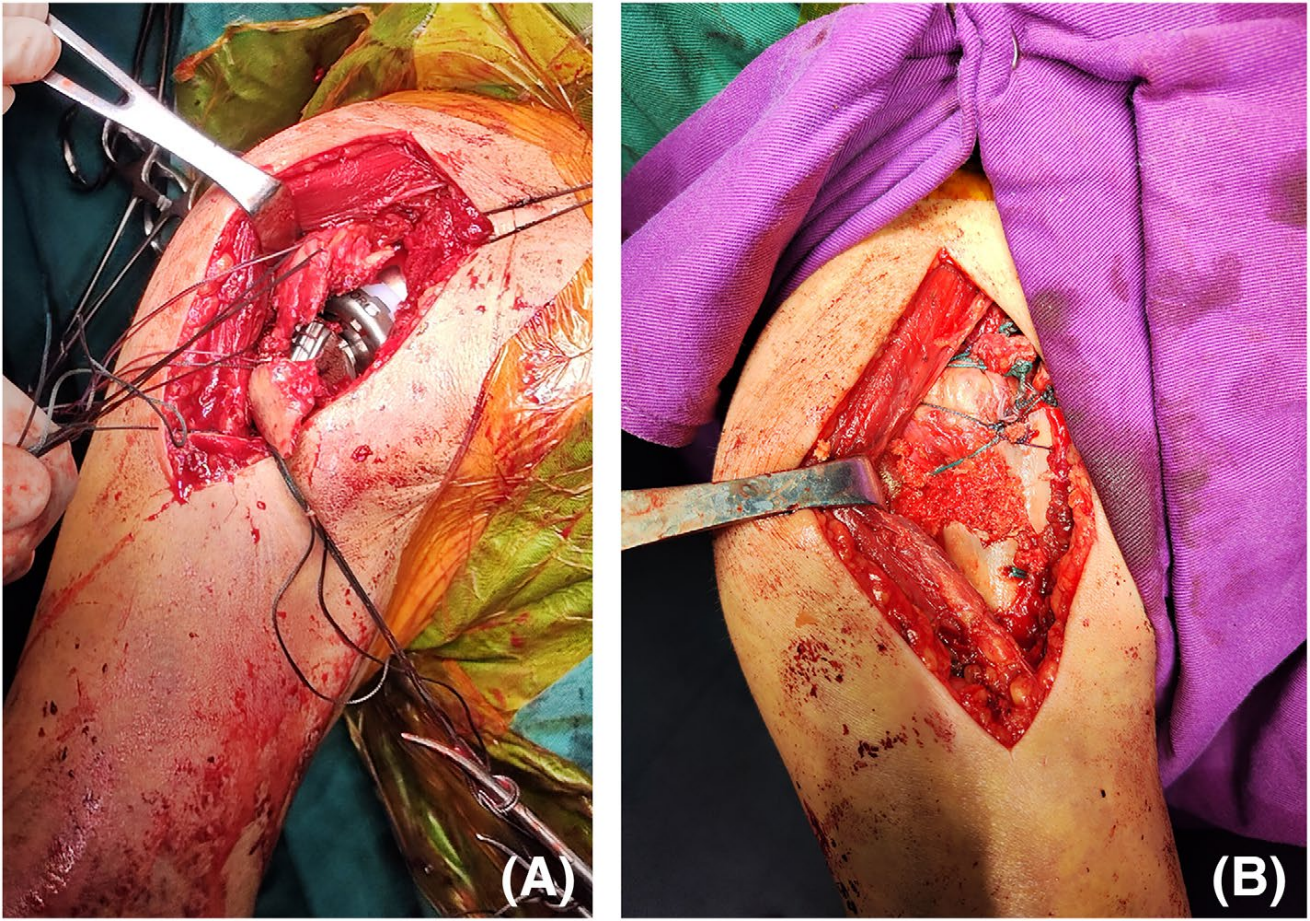
Main steps of surgery. (A) The prosthesis was implanted; (B) Fix the greater and lesser tuberosities to the proximal humerus.

All stem components were cemented in place. The posterior tilt angle of the prosthesis was standardized at 20°. Following a trial reduction, adjustments to the humeral version and length were made to evaluate joint laxity, stability, and acromial impingement. The humeral tray and bearing were subsequently assembled. Post‐reduction, the final ROM and muscle tension were assessed (Figure 2A). Tuberosities in the primary RSA group were reduced and fixed to the stem using No. 5 Ethibond sutures, with cancellous bone grafting to foster bone union (Figure 2B). In the salvage group, the original configuration of the tuberosities was preserved, and no osteotomy was conducted to correct malunion. The long head of the biceps tendon was sutured to the conjoint tendon. A drainage system was utilized for the immediate postoperative period.

### 2.3 Postoperative Management

All patients received a standard antibiotic regimen for 72 h postoperatively. Drains were typically removed within 1–2 days following surgery. A sling was utilized for immobilization of the shoulder for 6 weeks after surgery. Initiation of passive movement exercises of the shoulder commenced 6 weeks postoperatively, with progression to active exercises by 8 weeks post‐surgery. At the three‐month mark, patients advanced to higher‐intensity muscle‐strengthening exercises.

Standard anteroposterior and lateral radiographs were obtained for all patients 1–2 days after surgery to assess the positioning of the prosthesis.

### 2.4 Evaluation Criteria

Functional outcomes were evaluated preoperatively and at 6 weeks, 3, 6, and 12 months postoperatively, with ongoing assessments at regular annual intervals. For patients with acute PHF, preoperative ROM assessments were not feasible due to pain. Baseline subjective outcomes were documented based on preoperative medical records. Clinical outcomes were assessed using the Constant Score, American Shoulder and Elbow Surgeons (ASES) score, Visual Analog Scale for Pain (VAS), and range of motion (ROM) both pre‐ and postoperatively. Additionally, radiographic examinations were conducted for all patients to evaluate the status of the prosthesis.

#### 2.4.1 ROM

The ROM was assessed in terms of AF, ER, and IR with the arm at the side at each follow‐up visit, utilizing a goniometer for measurement.

#### 2.4.2 VAS for Pain

The VAS score is a psychometric instrument commonly employed in pain assessment to gauge the severity of pain experienced by patients. It typically consists of a 10 cm horizontal line, with higher scores indicating increased pain levels. Patients rate their pain intensity on a continuous scale from “no pain” to the “worst possible pain.”

#### 2.4.3 Constant Score

The Constant score is a widely used 100‐point scale designed to evaluate shoulder function. It encompasses subjective elements such as pain and activities of daily living (ADL), contributing 35 points, and objective measures including ROM and strength, accounting for 65 points.

#### 2.4.4 American Society of Shoulder and Elbow Surgery (ASES) Shoulder Joint Score

The ASES score is a comprehensive 100‐point metric assessing shoulder function in the context of various pathologies. It evaluates two dimensions—pain and performance in ADL—with each domain representing 50 points of the total score. The pain domain addresses nighttime pain, analgesic usage, and self‐reported pain intensity. The ADL domain comprises 10 shoulder‐specific functional items, including toileting and hair combing.

#### 2.4.5 Radiographic Evaluation

Postoperative radiographic assessments were performed on all patients to evaluate the prosthesis status. Radiolucency at the component interface exceeding 2 mm in width is indicative of loosening. Other signs of loosening include changes in prosthesis position, linear osteolysis, and focal osteolysis observed on follow‐up radiographs [22].

### 2.5 Statistical Analysis

The statistical analyses were conducted using SPSS version 25.0 (IBM Corp., Armonk, NY, USA). Continuous variables such as age, follow‐up duration, operative time, blood loss, ROM, and postoperative scores (VAS, Constant, ASES) were described as mean ± standard deviation. These variables were analyzed using independent t‐tests or Mann–Whitney U tests based on data distribution. Categorical variables such as gender and Neer classification were described using frequencies. Categorical variables were analyzed using chi‐square tests or Fisher's exact tests. A *p* value of less than 0.05 was considered statistically significant.

## 3 Results

### 3.1 General Results

A total of 42 patients met the inclusion criteria, including 28 undergoing primary RSA for acute PHF and 14 undergoing salvage RSA for fracture sequelae. Demographic comparisons between the two groups are detailed in Table 1. Each patient was followed for a period exceeding 2 years, averaging 56 months in duration (range, 24–106 months). Table 1 presents the preoperative demographic characteristics of the participants in both groups.

**TABLE 1:** Patient demographics (mean ± standard deviation).

Variables	Primary RSA (*n* = 28)	Salvage RSA (*n* = 14)	t/χ^2^	*p*
Sex (M/F)	7/21	5/9	0.525	0.469
Age (years)	73.8 ± 4.5	62.1 ± 12.3	4.485	0.000
Follow‐up, months	55.2 ± 37.3 (range, 26–98)	49.5 ± 18.9 (range, 36–72)	1.728	0.087
Neer classification			0.223	0.782
3 part	8	5		
4 part	20	9		
Blood lose, mL	389.7 ± 128.2 (range, 200–600)	430.4 ± 308.9 (range, 200–800)	−0.473	0.011
Operative time, min	150.2 ± 28.3 (range, 110–180)	188.1 ± 41.6 (range, 120–240)	−3.070	0.023

No significant differences in gender distribution and follow‐up period were observed between the two groups. The distribution of Neer classifications for PHF was similar (Table 1). Patients in the salvage RSA group were significantly younger than those in the primary RSA group (62.1 ± 12.3 vs. 73.8 ± 4.5 years, *p* < 0.001). The operative time was longer for the salvage group compared to the primary group.

In the fracture sequelae group, the etiologies included 5 cases of humeral head osteonecrosis, 4 cases of nonunion, 2 cases of malunion, 1 case of postoperative infection, and 2 instances of rotator cuff failure following ORIF. The mean interval from the initial injury to the salvage RSA was 8.5 months (range, 6–16 months).

### 3.2 Clinical Outcomes

#### 3.2.1 ROM

At the final follow‐up, the mean AF, ER, and IR in the primary group were 115.9° ± 29.1°, 28.8° ± 14.1°, and 7 ± 2, respectively. In the salvage group, these measurements were 101.4° ± 52.3°, 26.4° ± 16.41°, and 7 ± 2, respectively. No significant differences in AF, ER, or IR were observed between the two groups (Table 2).

**TABLE 2:** Comparative postoperative outcomes (mean ± standard deviation).

Variables	Primary RSA (*n* = 28)	Salvage RSA (*n* = 14)	*t*	*p*
AF (°)	115.9 ± 29.1	101.4 ± 52.3	1.156	0.436
ER (°)	28.8 ± 14.1	26.4 ± 16.4	0.475	0.762
IR	7 ± 2	7 ± 2	−0.729	0.762
VAS	1.2 ± 1.5	1.6 ± 1.9	−0.866	0.607
Constant	79.4 ± 15.9	74.1 ± 23.3	0.873	0.762
ASES	84.0 ± 13.8	81.9 ± 15.4	0.441	0.535

In the salvage group, AF significantly improved from 50.5° ± 22.4° preoperatively to 101.4° ± 52.3° postoperatively (*p* < 0.05). Similarly, ER showed a statistically significant increase from 21° ± 15.2° preoperatively to 26.4° ± 16.41° postoperatively (*p* < 0.05). No significant change in IR was noted (Table 3).

**TABLE 3:** Clinical outcomes after RSA compared with those before RSA in the salvage group.

Variables	Preoperation	Postoperation	*t*	*p*
AF (°)	50.5 ± 22.4	101.4 ± 52.3	3.457	0.000
ER (°)	21 ± 15.2	26.4 ± 16.4	2.123	0.017
IR	6 ± 3	7 ± 2	1.037	0.924
VAS	6.2 ± 2.5	1.6 ± 1.9	−4.568	0.023
Constant	37.2 ± 11.5	74.1 ± 23.3	5.310	0.002
ASES	36.1 ± 12.2	81.9 ± 15.4	8.720	0.001

#### 3.2.2 VAS Score for Pain

Postoperative pain decreased significantly in both groups (*p* < 0.05). The primary group demonstrated a reduction in VAS scores from 6.8 to 1.2, while the salvage group showed a decrease from 6.2 to 1.6. No significant difference in postoperative pain was observed between the two groups (Tables 2 and 3).

#### 3.2.3 Constant Score

At the final follow‐up, the primary group scored 79.4 ± 15.9 versus 74.1 ± 23.3 in the salvage group. No significant differences in Constant scores were noted between the two groups (*p* = 0.723) (Table 2). Within the salvage group, the Constant score showed a significant improvement from 36.1 ± 12.2 (ranging from 15 to 48) preoperatively to 74.1 ± 23.3 (ranging from 19 to 98) postoperatively (*p* < 0.05; Table 3).

#### 3.2.4 American Society of Shoulder and Elbow Surgery Shoulder Joint Score (ASES)

**FIGURE 3:**
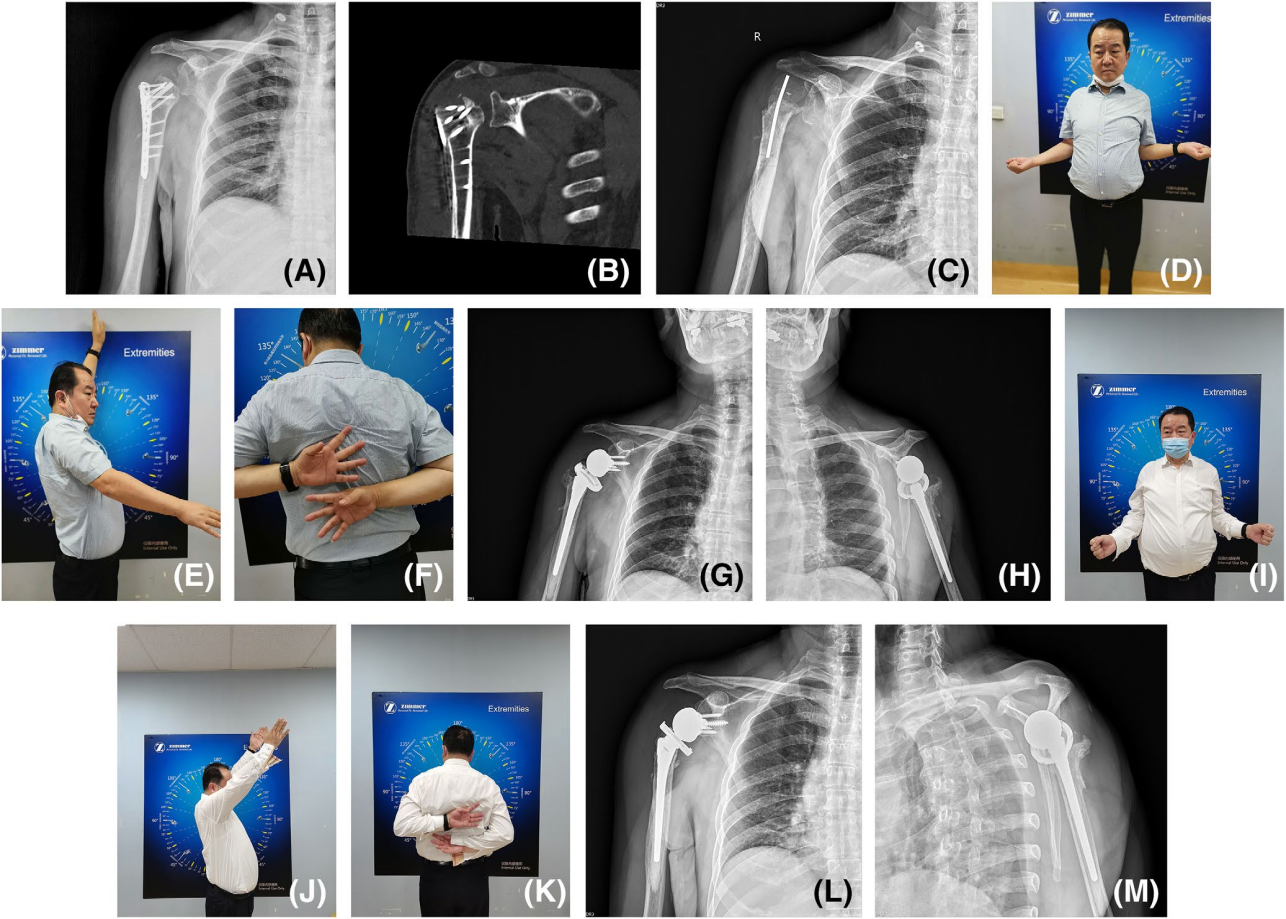
Representative case of a 53‐year‐old male with post‐ORIF infection and proximal humeral bone loss. (A, B) Radiographic examinations showed a severe bone defect of the proximal humerus. (C) A thorough debridement and antibiotic cement spacer were introduced to the shoulder joint. (D–F) Functional limitations prior to salvage RSA. (G, H) A two‐stage revision RSA was performed for the patient. The postoperative X‐rays showed that the prosthesis position was satisfactory after RSA. (I–K) 6 months after surgery, the forward elevation was 140°; external rotation was 30°; IR was T12 level. VAS score was 0; The ASES score was 88; Constant‐Murley score was 89. (L, M) 14 months after surgery, the X‐rays showed no signs of loosening.

No patient in either group exhibited signs of shoulder instability. The average postoperative ASES scores for the primary RSA and salvage RSA groups were 84.0 ± 13.8 points (range, 57–100 points) and 81.9 ± 15.4 points (range, 42–100 points), respectively. No significant differences in ASES scores were observed between the two groups (Table 2). In the salvage group, the ASES score significantly improved from 37.2 ± 11.5 points (range, 28–55 points) preoperatively to 81.9 ± 15.4 points (range, 42–100 points) postoperatively (*p* < 0.05) (Table 3). Typical cases are shown in Figure 3.

#### 3.2.5 Radiographic Results

The final follow‐up radiographic evaluations revealed no signs of notching of the inferior scapular neck in any of the cases. In the primary RSA group, migration or absorption of the greater tuberosities of the humerus occurred in six cases during the follow‐up period.

#### 3.2.6 Complications

Suture removal was uneventful at 2 weeks post‐surgery, with no incision‐related complications. No cases of prosthetic joint infection, joint instability, acromial stress fracture, or dislocation were reported at follow‐up.

Primary RSA Group: In the primary RSA group, one patient sustained a minor periprosthetic humeral fracture intraoperatively, which was successfully managed with 5# Ethibond non‐absorbable sutures. No subsequent instability or periprosthetic fractures were detected upon follow‐up in this patient. No revisions or reoperations occurred.

Salvage RSA Group: In the salvage RSA group, one patient developed a periprosthetic humeral fracture, and another patient exhibited aseptic loosening; both cases underwent revision surgery. Additionally, one patient in the salvage group presented with symptoms of an axillary nerve lesion but subsequently recovered following a 5‐month course of neurotrophic drugs. The major complication rate in the salvage group was 14.3%, which was notably higher than that observed in the primary RSA group.

## 4 Discussion

In this retrospective study, we compared the clinical outcomes of patients who underwent primary RSA for acute PHF with those who received salvage RSA for fracture sequelae. Our primary findings indicate that RSA is an effective treatment for PHF, with comparable clinical and radiographic outcomes observed in both groups over a mean follow‐up period of more than 4 years. However, salvage RSA was associated with a significantly higher complication rate (14.3% vs. 3.6%), consistent with previous literature [23] while highlighting the need for careful patient selection and surgical technique.

### 4.1 Efficacy of RSA for PHF

Displaced PHFs, especially in the elderly, present a clinical challenge due to the prevalence of osteoporosis. Despite a range of treatment options including conservative management, open reduction and internal fixation (ORIF), and prosthetic replacement, there is no universal consensus on the optimal treatment [24]. ORIF with a locking plate is commonly considered to be the first‐line treatment, but some severe postoperative complications have been reported [25]. The increasing incidence of PHF, especially among younger patients, has heightened awareness of these complications. In recent years, RSA has emerged as a popular treatment for complex, acute PHF in the elderly [26]. Prior research has reported satisfactory outcomes following RSA, with Klein [27] documenting ASES and Constant scores of 68 in a series of 20 patients. Lenarz [12] reported an ASES score of 78 among 30 patients. A systematic review [28] corroborated these findings, highlighting RSA as an effective treatment for acute PHF, with an average forward flexion of 118°, an average external rotation of 20°, and an average ASES score of 64.7. The functional outcomes (mean ASES score: 84.0 ± 13.8; Constant score: 79.4 ± 15.9) and ROM (mean AF, ER, and IR: 115.9° ± 29.1°, 28.8° ± 14.1°, and 7 ± 2) of primary RSA in our cohort are consistent with prior studies [12].

Our findings, which demonstrate improvements in ASES and Constant scores post‐RSA for acute PHF, affirm that RSA yields reliable clinical outcomes. These outcomes are characterized by a favorable ROM, VAS scores, Constant scores, and ASES scores.

### 4.2 Outcome of RSA as a Salvage Option for Fracture Sequelae

The management of fracture sequelae following PHF is particularly challenging, with limited studies addressing this issue. Salvage treatment options are typically restricted to hemiarthroplasty, total shoulder arthroplasty, and RSA. Diverse clinical outcomes have been reported for hemi‐and total shoulder arthroplasties in the revision of such sequelae [29, 30]. Currently, there is scant information regarding the efficacy of RSA in addressing fracture sequelae. However, existing studies suggest that RSA offers superior clinical outcomes and lower rates of major complications compared to hemiarthroplasty, especially with a follow‐up period of at least 5 years [31].

Some studies have indicated that RSA can achieve satisfactory clinical results in the treatment of fracture sequelae, but with a higher rate of complications [16]. These complications often arise due to anatomical distortions [32, 33]. Nonetheless, other studies have reported an absence of major complications or reoperation following RSA for PHF sequelae [15, 34].

The results of our study suggest that salvage RSA also yielded significant improvements in pain relief (VAS: −4.6) and functional scores (Constant: + 36.9; ASES: + 45.8), mirroring findings by Martinez et al., who noted similar gains in patients with posttraumatic sequelae [20].

### 4.3 Controversy of Salvage RSA for Fracture Sequelae

Salvage RSA as a revision surgery for failed ORIF has been reported to carry a high rate of complications, including infection and loosening [1]. Reoperation rates for salvage RSA have been cited as high as 40%, primarily due to early postoperative infections, while the incidence of aseptic loosening and recurrent instability requiring revision has remained relatively low [28]. Generally, salvage arthroplasties for fracture sequelae are associated with inferior outcomes and higher complication rates compared to primary arthroplasties [9, 15]. Nonetheless, some studies have reported more favorable outcomes for RSA over anatomic shoulder arthroplasty in the revision of fracture sequelae [34, 35]. Additionally, significant improvements in functional outcomes and patient satisfaction have been documented in the majority of patients who have undergone salvage RSA [17, 32]. RSA appears to be the optimal choice for managing most fracture sequelae, particularly in cases of severe shoulder deterioration. A particular study by Black et al. [36] noted RSA's effectiveness in reducing pain and improving function in younger patients (mean age 59.3 years) who had undergone failed arthroplasty, with satisfactory functional outcomes and no major complications observed over a follow‐up period of more than 4 years. Despite these findings, the available data on the clinical and radiographic outcomes following primary RSA and salvage RSA remain limited.

The present study contributes to the body of evidence by retrospectively comparing outcomes of primary RSA with those of salvage RSA for fracture sequelae. Our findings indicate that salvage RSA can yield satisfactory outcomes for patients with fracture sequelae, with results comparable to those of RSA for acute fractures. Our results diverge from some earlier reports. For instance, Nelson et al. found lower Constant scores in salvage RSA compared to primary procedures (mean difference: −10 points, *p* = 0.03), whereas our cohort showed no significant intergroup difference (79.4 vs. 74.1, *p* = 0.723) [1]. This discrepancy may reflect differences in surgical technique or sample size limitations. Conversely, Black et al. observed a 40% reoperation rate in younger salvage RSA patients, far exceeding our 14.3% rate, likely due to stricter patient selection and senior surgeon involvement [36]. Such variability underscores the need for standardized protocols in revision settings. While the outcomes of salvage RSA in our study are promising, longer follow‐up periods are necessary to fully evaluate the clinical and radiographic outcomes of these patients.

### 4.4 Complications and Prevention

Previous studies reported that the complication rate of the primary RSA for acute PHF was 9%–17% [19, 22, 30]. Some studies have reported salvage RSA with a higher rate of complications [1, 37]. A systematic review reported that the overall complication rate in the salvage RSA groups was 18.7%, and the most common complications included dislocation, periprosthetic fracture, infection, implant loosening, cortical perforation, and component malposition [1]. Prior surgery would result in anatomic distortions and soft‐tissue scarring [32, 33], which would cause technical errors in revision surgery. On the other hand, Black reported a comparable complication rate of salvage RSA compared with primary RSA [36]. A previous study has reported the complication rate of salvage RSA as high as 68%; however, the complication rate would be greatly decreased by a senior surgeon who has performed more than 40 cases of RSA [38]. We believe most of the complications can be avoided by thorough preoperative planning and delicate surgical manipulation. Raiss [39] reported a dislocation rate of up to 34% for salvage RSA. As a previous study reported [20], we think that dislocation was due to excessive soft tissue release and the bone loss of the proximal humerus, which makes it more difficult to correct the position and height of the prosthesis.

In our study, all the greater tuberosities of the humerus were accepted as they originally were during salvage RSA, and no osteotomy was performed, which was reported to have a higher complication rate and worse shoulder function [30]. In addition, some surgeons have also recommended that the morphology of the tuberosities should be kept for any case, and no osteotomy was needed to transform malunion for salvage RSA [1, 29, 37]. Because the shoulder function of patients who underwent RSA is mainly supported by the deltoid muscle instead of glenohumeral joint integrity or rotator cuff function [1].

Although salvage RSA can significantly improve function, the higher complication rate cannot be ignored. A recent retrospective analysis based on health insurance data indicated that the complication rate of salvage RSA (18.7%) was significantly higher than that of primary RSA (9.2%), mainly attributed to anatomical destruction and soft tissue scar formation [23]. In this study, the complication rate of 14.3% in the salvage group was more manageable compared to the 34% dislocation rate reported by Raiss et al., which may be attributed to the preservation of the integrity of the greater tuberosity during surgery and accurate prosthesis positioning [37]. However, the risk of infection still needs to be monitored. Previous studies have reported an infection rate ranging from 3% to 11% [40]. In this group of cases, no cases of infection were observed, which was achieved through thorough debridement and staged surgery [35, 40]. In our study, no infections were observed, which is mainly attributed to thorough joint debridement. Besides, our routine antibiotic prophylaxis was administered 30 min before the skin incision with first‐generation cephalosporin and lasted for 72 h postoperatively.

Our study revealed that RSA as a primary and salvage option for PHF can achieve satisfactory outcomes but should be used with extreme caution, especially in revision surgery. Similar to other reports, the complication rate in this study was higher in the salvage group.

### 4.5 Surgical Tips

Based on our findings and existing evidence, we propose the following recommendations: Achieving rigid fixation between bone fragments of tuberosities and the humeral stem is paramount to prevent rotational instability. This involves securing bone fragments with horizontal and vertical sutures to the proximal stem and humeral shaft. Interpositional cancellous bone grafting between the tuberosities and humeral stem reduces displacement risks in osteoporotic patients, enhancing bony union rates. Salvage RSA should prioritize patients with severe bone loss or rotator cuff deficiency, avoiding active infections or neurovascular compromise [29, 34]. Meticulous debridement of scar tissue is performed while preserving deltoid integrity. Intermittent retractor relaxation minimizes traction‐induced neurovascular injury, a common pitfall in revision settings. Retaining the native tuberosity morphology without corrective osteotomy simplifies implantation and maintains deltoid tension, which is critical for postoperative stability [29]. Preserving tuberosity integrity, avoiding excessive soft tissue release, and using 3D‐CT for implant positioning may reduce dislocation risks [16, 39]. Layered closure of joint cavity dead space and soft tissue coverage over the prosthesis mitigates infection risks, particularly in two‐stage revisions for prior infections [40].

## 5 Limitations and Strengths

Our study has several limitations. Firstly, its retrospective design introduces potential biases. Secondly, the follow‐up periods, while adequate for this analysis, may be considered brief in the context of arthroplasty, necessitating longer‐term monitoring for implant failure and other late complications. Thirdly, the relatively small sample size, especially in the salvage group, may restrict the generalizability of our findings and potentially affect the statistical power to detect significant differences between groups. This limitation should be considered when interpreting the results, as it may introduce bias and reduce the reliability of our conclusions.

While our study presents certain limitations, it also possesses notable strengths. Firstly, our study offers a detailed comparative analysis of primary and salvage RSA, a comparison that is scarce in the current literature. Secondly, our long‐term follow‐up period of over 4 years provides valuable insights into the durability of RSA outcomes in both acute and sequelae settings. Thirdly, the study's comprehensive evaluation of clinical outcomes, including ROM, pain scores, and functional assessments, contributes to a holistic understanding of patient recovery post‐RSA.

## 6 Conclusions

Our findings indicate that salvage RSA for fracture sequelae achieves functional outcomes comparable to primary RSA for acute PHF in the early postoperative period, with significant pain relief and improved functional scores. However, the complication rate was higher in the salvage group, underscoring the need for careful patient selection, meticulous surgical technique, and vigilant postoperative care.

## Author Contributions

**Qing Zhang:** data collection, statistics, paper writing. **Zhou Xiang:** study design. **Ming Xiang:** operation implementation. **Sujan Shakya:** prepared figures. **Yi Cao:** data collection. **Xin Duan:** study design, data collection. All authors reviewed the manuscript. All authors have read and agreed to the published version of the manuscript.

## Ethics Statement

The study was conducted in accordance with the Declaration of Helsinki and approved by the Ethics Committee of Sichuan Provincial Orthopedic Hospital (grant number KY2023‐005‐01). Written informed consent was obtained from all subjects involved in the study.

## Consent

The authors have nothing to report.

## Conflicts of Interest

The authors declare no conflicts of interest.

## Data Availability

Original data supporting the results of this study are available from the corresponding author upon request if needed.
